# In Search of Trustworthy and Transparent Intelligent Systems With Human-Like Cognitive and Reasoning Capabilities

**DOI:** 10.3389/frobt.2020.00076

**Published:** 2020-06-19

**Authors:** Nikhil R. Pal

**Affiliations:** Indian Statistical Institute, Electronics and Communication Sciences Unit, The Centre for Artificial Intelligence and Machine Learning, Calcutta, India

**Keywords:** Artificial Intelligence, Deep Neural Networks, explainable AI, trustworthy AI, machine learning, sustainable AI, deep learning

## Abstract

At present we are witnessing a tremendous interest in Artificial Intelligence (AI), particularly in Deep Learning (DL)/Deep Neural Networks (DNNs). One of the reasons appears to be the unmatched performance achieved by such systems. This has resulted in an enormous hope on such techniques and often these are viewed as all—cure solutions. But most of these systems cannot explain why a particular decision is made (black box) and sometimes miserably fail in cases where other systems would not. Consequently, in critical applications such as healthcare and defense practitioners do not like to trust such systems. Although an AI system is often designed taking inspiration from the brain, there is not much attempt to exploit cues from the brain in true sense. In our opinion, to realize intelligent systems with human like reasoning ability, we need to exploit knowledge from the brain science. Here we discuss a few findings in brain science that may help designing intelligent systems. We explain the relevance of transparency, explainability, learning from a few examples, and the trustworthiness of an AI system. We also discuss a few ways that may help to achieve these attributes in a learning system.

## 1. Introduction

In this article, we focus on a few issues that need attention to realize AI systems with human-like cognitive and reasoning abilities. For some of these issues we are not in a position to suggest precise solutions, but in some cases we shall provide cues or pointers to areas where we may look for to find possible solutions.

### 1.1. Learning and Intelligence

We begin with a definition of learning. Oxford dictionary^1^ defines *learning* as “The acquisition of knowledge or skills through study, experience, or being taught.” It defines *intelligence* as “The ability to acquire and apply knowledge and skills.” The Cambridge dictionary^2^, on the other hand, defines intelligence as “the ability to learn, understand, and make judgments or have opinions that are based on reason.”

Thus, learning and intelligence are intimately related. In our view, judicious applications of the knowledge learnt lead to intelligence. Learning, in the context of machine, raises several fundamental questions: What to learn (extraction of knowledge), how to learn (representation), and how to use the same. Today to an AI researcher, learning usually means extracting knowledge from data (explicitly or implicitly) and then applying that knowledge to make decisions. Here the word “data” has been used in a broader sense, where data can come from observations/measurements or from human beings. Some desirable attributes of an intelligent system are: ability to learn (from data/experience), understand, make judgment, reason, and apply what has been learnt to unknown situations. It should also know when to refrain from making a judgement.

### 1.2. Deep Learning and Artificial Intelligence

At present Deep Learning (DL) (LeCun et al., 1998; Krizhevsky et al., 2012) is probably the most successful vehicle for designing AI systems. It is fantastic in learning from data and making decisions but it is almost like a black box - no transparency. We shall see that these systems may fail miserably in cases where other systems possibly would not.

But what is deep learning? Before defining DL, it is probably natural to introduce what Machine Learning is. Arthur Samuel in 1959 coined the term “Machine Learning” (Samuel, 1959) in the context of a machine playing the game of Checkers. He wrote a checker program that could play against a human player as well as it could play against itself. Consequently, the program could play many games in a short time and by this process could learn the game better than its opposition. This gives us some idea of what machine learning is. More recently, Mitchell in his book “Machine Learning” (Mitchell, 1997) gave a formal definition of (Machine) Learning: “A computer program is said to learn from experience E with respect to some class of tasks T and performance measure P, if its performance at tasks in T, as measured by P, improves with experience E.” In the checker program of Samuel (1959), T is playing of checkers, P is percentage of times the ML system wins, E is either playing with itself or with a human, which generates a sequence of moves (data or experience). Similarly, for a machine learning system to classify images we can have: T as the task of classification, P as the misclassification error or square loss or cross-entropic loss, and E as a set of labeled image data. Equivalently, we can say that an ML system finds a function in a given class of functions (defined by the associated learning model such as neural network, a decision tree, a polynomial regression function) to fulfill a given purpose (say the task of classification) as best as it can (typically optimizing some criterion) based on experience (typically using a given set of training data). With this introduction to ML, we get back to Deep Learning. The basic of idea of DL is to learn complex concepts in terms of a hierarchy of concepts, where each concept in the hierarchy is defined in terms of its immediate lower level simpler concepts. The hierarchical organization allows representation of complicated concepts in terms of simpler concepts. If this hierarchical architecture is represented using a graph, the graph would be deep and hence it is called deep learning (Goodfellow et al., 2016). At present most DL systems are primarily based on multilayered neural network architectures. The way a Deep Neural Network (DNN)/ Convolutional Neural Network (CNN) works does not seem to have a strong relation to the way a human brain works. We shall discuss this issue later. However, DNN seems to enjoy a few properties: a large neural network with many layers, uses hierarchies of representation/abstraction, and gets better results with bigger models and bigger training data. Often such models have more free parameters than would be required to solve a given problem. This may lead to overfitting/memorization resulting in poor generalization. In order to increase the robustness of the system and to achieve better generalization to unseen data, various regularization techniques such as norm (*L*_1_ or *L*_2_ or both) of the weight parameters, dataset augmentation adding noise, drop outs, early stopping, and parameter sharing are used (Goodfellow et al., 2016).

### 1.3. Are We on the Right Track?

After the success of deep learning in many areas like image recognition and games (defeating Se-dol Lee, the best human GO player, by Google DeepMind's AlphaGo), often deep learning is viewed as an all-cure solution. Sometimes we get an impression that today AI is almost synonymous to DL. A Google search with ‘Neural Networks” retrieves about 30,800,000 results (April 17, 2020), “Artificial Intelligence” brings About 127,000,000 results, while “Deep Learning” brings About 39,800,000 results. It clearly reveals the rapid growth of interest in deep learning. It is worth noting that all pages with “neural networks” may not be related to artificial neural networks. It cannot be questioned that DL is one of the most successful tools to realize AI systems in some specific areas. Although DNNs have demonstrated unbeatable performance in some applications, DDNs are found to recognize images completely “unrecognizable to humans” as human recognizable objects with a very high confidence (Nguyen et al., 2015). At the other extreme, with very minor changes in an image that are imperceptible to human eyes, DNNs are found to mislabel the image (Papernot et al., 2017). It raises a big question. Why? A DNN or any other intelligent system usually optimizes some objective functions with respect to a set of learnable parameters. Such a system usually can make correct decisions. But for DNNs (and many others) neither the architecture nor the decision making rule can help to explain the rational for such a decision in terms of human understandable knowledge. Although we know precisely the computation done at different nodes, the system is not transparent because we do not know the semantics associated with each node or a group of nodes or the presence of causal relations, if any, between the nodes. It behaves like a black box. Moreover, while designing a DNN often the principle of Minimum Description Length (MDL) is not given its due importance, as a result a system may have more degrees of freedom than what is required, which may lead to inappropriate generalization. Given a finite data set, the MDL principle suggests to pick the most compact description (parameterization) of the model as well as the description of the data under that model. However, the current deep networks are far from that. For example, the VGG 19 network has about 150 M parameters (Canziani et al., 2016). One of the possible reasons is that small networks cannot be trained with the present available techniques to achieve similar performance for large scale classification problems. However, large number of free parameters increases the expressibility of such networks and they can easily memorize data. For example, Zhang et al. (2017) demonstrated that networks like Inception and Alexnet can learn/memorize almost perfectly the CIFAR10 dataset with random permutation of labels. Of course, the test accuracy is no better than random chance. Another related important issue is that these systems are not equipped to deal with the open world nature of most decision making problems. In order to realize a transparent, accurate, and trustworthy system with human like reasoning abilities, in our view, there are few areas that we may need to focus on. Next we discuss a few such issues/areas.

## 2. What's Next?

### 2.1. Look Into the Brain

The human brain is one of the most complex systems in the known universe. In spite of tremendous efforts, our understanding about how brain learns is quite limited, yet a good amount is known. In order to have a learning system with reasoning abilities like a human being, in our view, we should try to exploit our knowledge from the brain science. The more we can mimic the brain, the higher is the chance that we shall be able to realize human-like attributes in an AI system. For example, functional imaging studies with cellular resolution (*in vivo* two-photon calcium imaging) revealed that in area 18 of cat visual cortex there are extremely ordered groups of neurons that respond to visual stimuli of a particular orientation. Neurons having opposite directional preferences for visual stimuli were separated with a high spatial precision in three dimension (Ohki et al., 2005). If we could explicitly incorporate such features in our computational NN, it would behave a little more like a biological system. To design a learning system, we can generalize this idea assuming that for different types of visual objects different clusters of neurons will respond. In this case, the network will be more transparent than the a conventional MLP. However, to the best of our knowledge, computational neural network models including DNNs do not exploit this, although, the response of some neuron or a group of neurons may be directionally oriented due to the training. In fact, it is possible to integrate the principle of self-organizing map along with a multilayered neural network so that different spatial clusters of neurons get activated by instances from different classes (Bandyopadhyay, 2018).

Usually the success of a class of DNNs is attributed to the unsupervised feature extraction, i.e., on the representation and abstraction of raw data. In a convolutional neural network (CNN), actually we compute cross-correlation and use max-pooling to achieve reduction of information with a hope to realize useful abstractions. There is no explicit cause-effect relation. But does it have anything to do with information processing in the brain of living beings? There are evidences supporting a complex and hierarchical feature representation along the ventral visual stream (DiCarlo et al., 2012; Kuzovkin et al., 2018). A CNN makes a hierarchy of complex feature representation for image recognition and from this point of view, a CNN has some similarity with the feature representation in a biological vision system. We recall here a remark in DiCarlo et al. (2012) “*We do not yet fully know how the brain solves object recognition.”* Recently, analyzing the intracranial depth recordings from human subjects researchers suggested that the gamma activity matches the increasingly complex feature representation/abstraction in a deep convolutional neural network. In reality, the information abstraction in a biological system is much more complex. For example, in a natural scene there is a high degree of spatial and temporal correlation, and hence, representation of visual information at the level of photoreceptors would be highly inefficient because of tremendous redundancy. Dan et al. (1996) conducted some experiments with cats. They used movies of natural scenes as inputs and the responses of single lateral geniculate nucleus (LGN) neurons were recorded using a single tungsten or a multi-electrode array. They analyzed the temporal correlation and power spectra of the responses, which demonstrated that the natural visual information is temporally de-correlated at the lateral geniculate nucleus. Each LGN has six layers of neurons; it receives input from the retina and sends information to the primary visual cortex. Consequently, if an image recognition system exploits these concepts at the feature extraction stage, it would resemble more a biological system and is likely to yield better results. There have been several attempts to develop computational models of LGN neurons (Einevoll and Halnes, 2015; Sen-Bhattacharya et al., 2017). We need to investigate how these models can be adapted to develop machine learning systems for computer vision.

The primary visual cortex, the visual area 1 (V1), uses sparse code with minimal redundancy to efficiently represent and transmit information about the visual world and the non-classical receptive filed plays an important role in this process (Vinje and Gallant, 2000). Sparse modeling is also used to identify “Sparse Connectivity Patterns” (SPCs) which make a parsimonious representation of brain functions (Eavani et al., 2015). At the level of neurons, we have sparse codes and at a higher level, we have these SPCs, which represent different system-level functions and relate to a set of spatially distributed, functionally synchronous brain regions. It is also known that the processing in the brain is distributed. In fact, an important characteristic of the brain is believed to be sparse distributed representation (SDR) (Ahmad and Hawkins, 2016; Hawkins, 2017; Pal, 2018a). In an SDR, at a given instant of time only some of the neurons may be active (producing an output of 1). If two SDRs have some overlap (have some common active neurons) then the two SDRs share some common attributes of the two concepts. The SDR characteristic is considered very important for biological intelligence. Since SDR makes an efficient representation of information and plays a key role in biological intelligence, incorporation of such ideas in designing learning systems is expected yield better AI systems.

### 2.2. Learning From a Small Sample

One of the claimed advantages of DNN is “more is better”—if we can train a big network with more data we can get a better performance. But does a human being need thousands of images to distinguish between various objects? Even a baby can learn to distinguish between a large number of animals with just a few presentation of the animal images. More importantly, once a human learns the concept of “animals”, given a picture of a completely strange animal that has never been seen by the person, he/she can easily detect that it is an animal and will never mistake it to be a car or a human. This is an extension of the learned information by “common sense.” So human learning must be doing something different or at least something additional than what DNNs or other ML systems do. A child can learn many objects within a short time with few examples. We need to develop systems that can learn from a limited number/variety of examples as living beings do. Such a learning system should not need millions of examples with wide variations and a large number of cycling through the data. There have been a few attempts to learn a class from just one or a few examples (Fei-Fei et al., 2006; Maas and Kemp, 2009; Lake et al., 2011, 2013, 2015; Wang et al., 2019). This is known as Few-Shot Learning (FSL) (Wang et al., 2019). In FSL the learning is accomplished with a few instances with supervisory information for the target and it often exploits prior knowledge. In Lake et al. (2013), an interesting hierarchical Bayesian model based on compositionality and causality for one-shot learning has been proposed. Lake et al. (2013, 2015) combined principles of compositionality, causality, and learning to realize a Bayesian Program learning framework to learn visual concepts using just one example. This method can achieve human-level performance and has defeated two deep network models. Although such a method takes inspiration from cognitive science, it does not take into account how human brains learn concepts from just one or two examples. There have been a few more attempts to one-shot learning. Learning from limited examples is very important in areas like medical science where usually only a handful instances from the positive class are available. In such a case, the interactive machine learning (iML) with “human-in-the loop” could be very useful (Holzinger, 2016; Holzinger et al., 2016). There are three main advantages of iML: First, human intervention can reduce the search space drastically. Second, it can facilitate learning using a limited number of instances. Third, it can help to open the “black Box” to some extent. However, it has a few limitations also. For example, human knowledge often suffers from subjectivity and hence the resultant system may have subjective bias. Thus, an objective evaluation of such a system is difficult. Because of the incorporation of subjective knowledge, replicability of the system is also difficult. Interactive machine learning with human-in-the-loop appears to have good potential for learning with limited data but it needs more investigation. Wang et al. (2019) provides an excellent exposition of FSL explaining the advantages of FSL, the challenges associated with it, and how some of the challenges can be addressed.

There are theories suggesting that human beings recognize/learn images by segmenting them at deep concavity and then viewing the object as a set of simple geometric components characterized by attributes such as curvature, collinearity, and symmetry (Biederman, 1987). It is worth noting that this is consistent with the idea of SDR discussed earlier. This theory of recognition by components may be useful in learning from one or a few images. To a living being every image is not equally memorable and it is found that color or simple image statistics, or object statistics do not make an image memorable (Isola et al., 2011). However, semantic information of objects and scenes is found to make an image memorable - a human being is able to remember such an image by just seeing once. This could be an important clue to design a learning system that can learn concepts with one or a few examples.

### 2.3. Explainable/Comprehensible/ Transparent AI

Most computational learning algorithms including DL are “blind” in learning. They are good in decision making, but cannot explain why a decision is made. Time demands more emphasis on this aspect of learning. Using regularizers one can simplify (reduce the complexity) of a decision making system, which is good but it fails to bring the level of transparency that we would be happy with. We note here that transparency of an AI system is different from its ability to explain the rational behind a decision that it makes (explainability). Transparency refers to understanding of the semantics associated with the computation that goes on in the system. Comprehensibility/transparency is a fuzzy concept with grades of membership in [0,1]. For a black box system like an MLP the membership is zero while for a completely transparent system it is one. For example, a decision tree is highly transparent as along as the attributes are understandable properties and the depth of the tree is small. However, as the depth of the tree increases or if we use extracted features like the principle components, we start losing its comprehensibility. Yet, it will remain more comprehensible than, for example, a multilayer perceptron. Similarly, a fuzzy rule-based system is also transparent. A fuzzy rule based system uses rules of the form (Chen et al., 2012) : If the expression level of Gene X is HIGH and the expression level of Gene Y is MODERATE and the expression level of Gene Z is LOW then the patient is suffering from Neuroblastoma. Here HIGH, LOW and MODERATE are linguistic values that are modeled by fuzzy membership functions. As long as the number of antecedent clauses is small, such rules are human understandable. Even if such a rule involves many antecedent clauses, it is still more transparent than, for example, a DNN. This is so because a fuzzy rule models a small hyperellipsoidal volume in the input space and assigns data points falling in that volume to a particular class with different degrees. Because of this very nature of fuzzy reasoning, we can easily understand how fuzzy rules work and why these are not likely to make an unexpected/unrealistic decision. However, machine learning tools like fuzzy systems or decision trees are poor performer compared to MLPs or DNNs. In particular, designing transparent decision trees or fuzzy rule based classifiers would be quite challenging when it comes to, for example, image recognition. One possibility may be to integrate a DNN and a fuzzy rule based system. We can use a DNN for feature extraction and abstraction. Then at the highest level we can use a fuzzy system for prediction or classification. This may add some level of transparency using the extracted features. However, we certainly need more. Another alternative may be to integrate experts' domain knowledge into the learning process or at the level of designing the system architecture.

The explainability problem can be approached at least in two different ways. First, by looking deeper into the trained AI systems to get some reasoning behind the decisions. Second, by using an additional layer or system to generate the explanations. For a decision tree or for a rule-based (fuzzy or crisp) system, generation of some explanation is relatively easy, but for a DNN, even for an MLP, it is quite difficult. A fuzzy rule-based system is naturally interpretable as it makes decisions based on fuzzy if-then rules (Hagras, 2018). These rules are easy to understand, for example, *If the body-temperature is HIGH and body-ache is SEVERE then the subject is suffering from flu*. Recently, there have been a few studies to explain why a DNN works. For example, researchers tried to discover which part of an image is primarily responsible to arrive at the final decision by the network (Simonyan et al., 2014; Zeiler and Fergus, 2014; Choo and Liu, 2018). There are other methods which use visual analytics to understand the learned representation and how it influences the output (Liu et al., 2017; Rauber et al., 2017; Choo and Liu, 2018). This kind of visual analytics are useful and help to understand a little better, but cannot make the system transparent or adequately explainable. Such tools/analytics cannot explain the reason behind a decision in a manner that a human would like to have. There have been other approaches to generate explanations for decisions made by a machine learning system (Hendricks et al., 2016; Ribeiro et al., 2016; Samek et al., 2017). For example, authors in Ribeiro et al. (2016) proposed a method to explain predictions made by any classifier by learning a local interpretable model around the prediction. On the other hand, Hendricks et al. (2016) proposed a method for generation of visual explanation for images classified by a deep network where the the explanations provide some justifications behind the classification and hence it is different from caption generation. The authors use a Long Short-Term Memory (LSTM) network (Hochreiter and Schmidhuber, 1997) along with the classifier. This is an interesting work but the explanation is generated by the LSTM. The big question of generating the explanation directly from the discriminator still remains. Very recent and useful expositions to the problem of explainable AI, its need and relevance, can be found in Hagras (2018), Goebel et al. (2018), and Holzinger (2018). Holzinger (2018) very nicely explains the advantages and limitations of automatic Machine learning (aML) and the advantages of iML. We have discussed earlier that a child needs only a few examples to learn different animals because humans can exploit the contextual information. Thus, we emphasize again that having a human-in-the-loop appears a very promising way of efficient learning. Holzinger (2018) also discussed a few promising approaches to realize explainable machine learning systems.

### 2.4. Recognize and React to the Open World Problems

Another consequence of blind learning is its failure to deal with the open world nature of recognition (not necessarily of visual information) problem. We have already mentioned that, DNNs sometime recognize images that are completely “unrecognizable to humans” as human recognizable objects with a very high confidence (Nguyen et al., 2015). Majority of the decision making problems are some kind of classification problems. Any decision making system should make recommendations based on only what it is taught in a broader sense. This does not mean that a learning system should not generalize, it must but to a “plausible/reasonable” extent. Let us clarify this. Suppose an AI system is trained to distinguish between tigers, lions and cows, where every animal in the training data set has four legs. Now if a cow, which has lost one leg in an accident is presented to the system, we expect the system to declare it as a cow with a reasonable level of confidence. But if the system is confronted with a dog or a goat it should simply say “I do not know.” Note that, here we are referring to systems for which we know why and when they should say “Don't know.” Most decision making systems including DNNs fail to respond properly when faced with known/unknown unknowns. Recognizing unknowns is very important for many applications such as medicine, healthcare, and defense. There are at least four problems related to this issue (Karmakar and Pal, 2018):

1. In an open-world situation, there are unknown classes (beyond the classes that a classifier is trained to classify). In this case, if a test data point comes from an unknown class, it will get wrongly classified.
2. In a closed-world situation, a test data point may come from one of the classes that the classifier is trained to classify, but it comes from outside the “sampling window” of the training data. In this case, the classifier will assign one of the trained classes and the assigned class may even be correct, but here the classifier should refuse to make any judgement.
3. In a closed-world scenario, we may get a test data point from outside the “sampling window.” In this case, the classifier may assign an unrealistic class. For example, the test data point may be located close to the training data of class *i*, but it is assigned to class *j*.
4. Even for a closed-world situation, there may be concept drift—the statistical characteristics of one or more classes may change with time. In such a situation, the classifier should not make any decision on the drifted data.

The above four problems are connected by a common thread; they arise when a test data point does not come from the sampling window of the training data. So, we can address all four problems if the machine learning system can detect and reject a point saying “Don't Know” when it does not come from the sampling window of the training data.

Many researchers have tried to address some of these problems (Chow, 1957, 1970; Dubuisson and Masson, 1993; Chakraborty and Pal, 2002, 2003; Scheirer et al., 2013, 2014; Jain et al., 2014; Karmakar and Pal, 2018). There have been quite a few attempts to deal with this problem, for example, using the Extreme Value Theorem (Scheirer et al., 2013, 2014; Jain et al., 2014). But such an approach suffers from a conceptual problem because known unknowns and unknown unknowns are not necessarily extreme values of the training data. In fact, these samples may be (usually will be) generated from a completely different distribution than that of the training data. Recently in Karmakar and Pal (2018) authors proposed a scheme to equip a multilayer perceptron network with the ability to say “do not know” when the test data come from outside the sampling window of the training data. In Karmakar and Pal (2018), a theoretically sound method for estimating the samapling window from a given training data set has been proposed.

Given the estimate of the sampling window, we can draw random samples from the complement of the estimated sampling window (of course over an slightly inflated hyper box containing the training data - any test data coming from outside this box can always be rejected) and use that to represent the “unknown world.” Now, for a c-class problem, we train a (*c*+1) class system, where the (*c*+1)^*st*^ class represents the “Don't Know” class. Further details can be found in Karmakar and Pal (2018). While training the system we may use suitable regularizer to control the sensitivity of the output for the (*c*+1)^*st*^ class with respect to inputs. This will help to train the system with limited samples from the “Don't know” class.

In principle, such a concept can be applied to DNNs also. But for very high dimensional data such methods would be computationally demanding. However, it may be possible to use appropriate regularizers to minimize the number of samples needed from the complement world for its faithful representation. In order to avoid occasional but catastrophic failure of an AI system, it must recognize its domain of operation beyond which it should not make any decision; otherwise, it may lead to situations giving a false perception that an AI system has “taken over the human.”

### 2.5. Trustworthiness of a Machine Learning System

**Figure 1:**
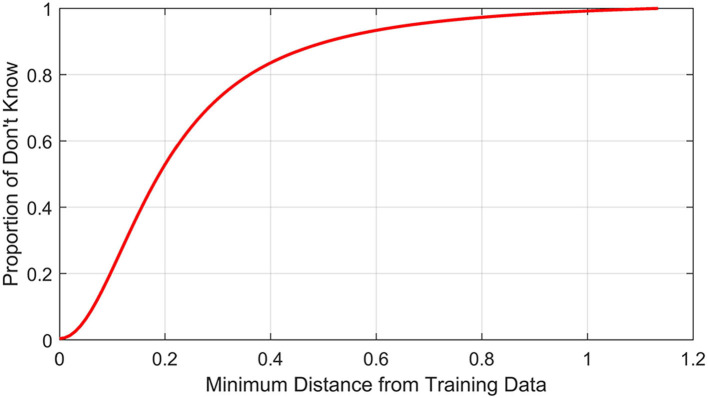
Illustration of how trustworthiness can be assessed.

When we use a so-called intelligent system either for medical diagnosis or for driving an unmanned vehicle, a natural question comes: how trustworthy the system is! To achieve trustworthiness we need to ensure two things: first, when test samples come from the sampling window, the system should not make wrong decisions and second, when the test data come from outside the sampling window, the system should refuse to make any recommendation (reject that point). To check the trustworthiness of such a trained system we may proceed as follows Karmakar and Pal (2018). We find the smallest hypercube containing the training data and then expand its each side by a small percentage, say 5%. We generate a large set of points uniformly distributed over the extended box. For each such point, we compute its shortest distance from the points in the training set. Let *D* be the largest value of all such distances. We divide the interval [0, *D*] into *k*>1 intervals. Let (*d*_*i*_, *d*_*i*_+Δ*d*);Δ*d*>0;0 ≤ *d*_*i*_ ≤ *D*; *i* = 0, 1, …, *k*; *d*_0_ = 0, *d*_*k*_ = *D, d*_*i*_ = *d*_0_+*i*.Δ*d*be one such interval. Let *N*_*i*_ be the number of points for which the shortest distance lies in (*d*_*i*_, *d*_*i*_+Δ*d*) and of these *N*_*i*_ points, *n*_*i*_ points are rejected by the network. Then, $f_{i}=\frac{n_{i}}{N_{i}};i=0,1,\ldots ,\left(k-1\right)$ is the percentage of points with distance in (*d*_*i*_, *d*_*i*_+Δ*d*) that are rejected. For a trustworthy system, with higher values of *i*, i.e., of *d*_*i*_, this percentage should increase. Thus, if we plot *f*_*i*_ vs. *d*_*i*_, for a trustworthy classifier, we expect to see a curve like the one in Figure 1. If *f*_*i*_ quickly goes to one with *d*_*i*_, this will suggest a very conservative and trustworthy system. Apart from the pictorial representation, it may be possible to come up with some index based on this curve to measure the trustworthiness of the trained system'. We just gave some idea of how to deal with this problem, but it certainly needs more focussed study.

### 2.6. Plausibility of Backpropagation

For majority of the learning algorithms, we use error backpropagation type approaches to optimize a learning objective. For the multilayer perceptron networks, the error backpropagation learning requires that the downstream errors are fed back to upstream neurons via an exact, symmetric copy of the downstream synaptic weight matrix. Thus, each neuron in the hidden layers requires the precise knowledge of all of its downstream connections (synapses). But it is believed to be almost impossible to happen in the brain (Lillicrap et al., 2016). Lillicrap et al. (2016) demonstrate that we can transmit teaching signal over different layers by multiplying errors with random synaptic weights and it becomes as effective as the backpropagation algorithm for training deep networks. This eliminates the strong structural constraint demanded by backpropagation making it a biologically more plausible architecture. Incorporation of such attributes in our NN would make the architecture of the system closer to that of a biological system. However, as clarified by Lillicrap et al. (2016) this does not answer more fundamental questions as to how exactly the brain computes and represents errors and how the feedforward and feedback paths may interact with each other.

### 2.7. Structure of Neurons

The neurons used in DNNs/CNNs are primarily of uniform structure and very simple in architecture. In biological vision, visual object recognition is realized by a hierarchical representation of increasingly complex features along the ventral visual stream (DiCarlo et al., 2012; Kuzovkin et al., 2018). This hierarchical representation (abstractions) are typical of deep learning. There have been several studies on establishing correspondence between the hierarchical representation of features with increasing complexity in the brain and that in CNN (Kriegeskorte, 2015; Yamins and DiCarlo, 2016). All these studies motivated researchers to investigate whether the neurons in our brain, which are responsible for thinking/reasoning and have much more complex tree-like structures with roots going deep in the brain and branches going close to the surface, can help to model computational neurons for realizing deep networks like CNN (Guerguiev et al., 2017). In the mammalian neocortex, feedforward and feedback signals are received at electronically segregated dendrites (Guerguiev et al., 2017). In order to realize a biologically more plausible neuron architecture for deep learning, Guerguiev et al. (2017) designed artificial neurons with two compartments, similar to the “roots” and “branches.” The hidden layer neurons are modeled to have segregated “basal” and “apical” dendritic compartments, which are used to separately integrate feedforward and feedback signals and the system did not require separate pathways for feedback signals. They demonstrated that such a network can easily learn to classify the MNIST data (LeCun et al., 1998) and it can also learn hierarchical abstract representation of the objects. This emphasizes as well as demonstrates that it is possible to design computational neurons which more closely model biological neurons as well as can make abstract representation of features like deep networks, and perform the task of classification. However, this does not necessarily mean that the brain replicates exactly this type of processing. Moreover, further investigations are needed to assess whether such networks are more robust or can perform reasoning more close to that by humans.

### 2.8. Artificial General Intelligence

We have indicated a few very minor cues from biology that may be useful, but there may be (actually there are) plenty of discoveries related to how brain stores, processes, and usees data/information to infer. We need to look at these areas if we want truly intelligent systems. In our view, to design AI systems with human-type reasoning, such concepts could be very useful for representation. For example, almost all of the successful AI systems of today primarily focus on only single task, say image recognition, and that too specific to a domain. If an AI system is trained to recognize natural scenes or animals, it usually cannot understand an X-ray image, summarize text information, or make medical diagnosis. If we want to realize a generalized AI system capable of doing multiple tasks, we may need to partition the network according to functionality of different lobes of the brain. Creating such an architecture and its training are certainly going to be challenging tasks. This will demand a better understanding of the brain and integration of various discoveries about the brain that we already know. In our view, a purely data-based design of AI systems, certainly is useful and will lead to many unexpected and successful applications, but it may not be adequate to realize true human-type cognitive and reasoning abilities.

## 3. Conclusion and Discussion

Any AI system, in fact any decision making system, should have a few desirable attributes. It should be accurate, transparent, trustworthy, simple, and be able to explain the decisions it makes. In addition, it would be good if the system can be trained with limited data with limited computation time. In our opinion, if we borrow ideas from the brain science to design decision making systems, we are *more likely* to realize human-like cognitive and reasoning abilities. We say “more likely” because we have mentioned earlier that our knowledge of how the brain learns is limited and the use of the partial knowledge to build an AI system, may not replicate brain style reasoning. Moreover, it may not always be an easy task to incorporate neuroscience discoveries into a computational AI system to realize the desired benefits. In this context we have discussed a few findings in brain science which can be exploited to design AI systems. We have also alluded how one can make a system trustworthy so that the system does not make a decision when it should not. We have provided some ideas on how we can quantify the trustworthiness of a system.

There are other important issues related to design of AI systems that have not been discussed here. For example, time has come to focus on sustainable AI (Pal, 2018b). Here we like to refer to two issues: The first issue is that the development (training) of the AI system should have the minimum carbon footprint. To achieve human-like performance often this important issue is ignored. To illustrate the severity of this issue we consider a recent study which used an evolution-based search to find a better architecture for machine translation and language modeling than the Transformer model (So et al., 2019). The architecture search ran for 979M training steps requiring about 32,623 h on TPUv2 equivalently 274,120 h on 8 P100 GPUs. This may result in 626,155 lbs of CO2 emission–this is about 5 times the lifetime average emission by an American Car (Strubell et al., 2019). The second point is that the solutions provided by an AI system should be sustainable with the minimum impact on the environment. For example, an AI system to assist farmers should not just try to maximize the yield, but should also keep in mind the impact of high use of nitrogen fertilizer on the environment. The system should prescribe the use of the Right nutrient source at the Right rate in the Right place and at the Right time (*R*^4^).

In near future, we shall see many remarkable advances in AI with many useful and innovative applications. In fact, time may come when just the accessability of the pages of a medical book by a computer would enable the system to scan the pages, understand them, extract the rules, and behave like a real doctor! Robots may interact with each other to redistribute work loads among themselves or repair each other's problems. AI applications will be almost everywhere and very intimately related to our daily life. Mostly there will be good usage but there may be some bad ones too. AI will lead to many legal issues also. We certainly will need global policies to monitor the use and abuse of AI.

The world renowned physicist, Stephen Hawking, commented during a talk at the Web Summit technology conference in Lisbon, Portugal,“Success in creating effective AI, could be the biggest event in the history of our civilization. Or the worst. We just don't know. So we cannot know if we will be infinitely helped by AI, or ignored by it and side-lined, or conceivably destroyed by it” (Kharpal, 2017). He also admitted that the future was uncertain.

It is not an easy task to equip any system (say a robot) with rules (based on data or otherwise) to deal with all possible scenarios for any non-trivial application. If such a robot is not explicitly trained to prevent itself from making decisions in unfamiliar situations, it may behave in an erratic manner and that may be viewed as if the robot has taken over the human. We believe, in near future AI systems will be extensively used almost everywhere and in some application areas (intentionally/unintentionally) uncontrolled behavior of robots may become a reality.

## Author Contributions

The author confirms being the sole contributor of this work and has approved it for publication.

## Conflict of Interest

The author declares that the research was conducted in the absence of any commercial or financial relationships that could be construed as a potential conflict of interest.

## Footnotes

^1^https://en.oxforddictionaries.com/definition/learning, June 2018.

^2^https://dictionary.cambridge.org/dictionary/english/intelligence, June 2018.
